# Prognostic factors in paediatric-onset multiple sclerosis: a narrative review

**DOI:** 10.1007/s00415-026-14029-z

**Published:** 2026-07-29

**Authors:** Mariaclara Achille, Massimiliano Copetti, Tommaso Guerra, Caterina Ferri, Pietro Iaffaldano, Sandra D’Alfonso, Maura Pugliatti, Roberto Palumbi, Marta Simone

**Affiliations:** 1 Child Neuropsychiatry Unit, Department of Precision and Regenerative Medicine, Jonic Area, University of Bari “Aldo Moro”, Bari, Italy; 2 Unit of Biostatistics, Fondazione IRCCS “Casa Sollievo Della Sofferenza”, San Giovanni Rotondo, Italy; 3 Department of Translational Biomedicine and Neurosciences (DiBraiN), University of Bari “Aldo Moro”, Bari, Italy; 4 Department of Neuroscience and Rehabilitation, University of Ferrara, Ferrara, Italy; 5 University of Piemonte Orientale “Amedeo Avogadro”, Novara, Italy; 6 Department of Clinical and Experimental Sciences, University of Brescia, Brescia, Italy

**Keywords:** Paediatric-onset multiple sclerosis, Disability progression, Prognostic factors, Disease-modifying therapy, Serum neurofilament light chain, Prediction models

## Abstract

**Background:**

Paediatric-onset multiple sclerosis (POMS) accounts for 3–10% of multiple sclerosis (MS) cases and differs from adult-onset disease in course, treatment response and long-term outcomes. Risk stratification is essential, yet the literature frequently conflates three distinct questions: which children develop MS (susceptibility), which convert from a first demyelinating event (conversion) and which, once diagnosed, will accrue disability (prognosis).

**Objective:**

This study aimed to provide a narrative overview of candidate prognostic factors in POMS, separated from susceptibility and conversion factors and organised by the outcome each predicts.

**Methods:**

PubMed and Google Scholar were searched (2002–2025, with hand searching of pivotal 2026 cohorts) using a predefined string and full eligibility criteria. Factors were classified along three axes of inference and graded into four evidence tiers (replicated; preliminary; susceptibility rather than prognosis; inconsistent). No formal risk-of-bias assessment was undertaken, consistent with the narrative design.

**Results:**

The most reproducible post-diagnosis predictors were early inflammatory activity (relapse number and inter-attack interval in the first two years, annualised relapse rate, early EDSS change), lesion topography (brainstem, spinal cord), T2 lesion accrual, serum neurofilament light chain and treatment-related variables (delayed disease-modifying therapy, early high-efficacy treatment). Advanced MRI metrics, serum glial fibrillary acidic protein and other fluid biomarkers remain preliminary; baseline EDSS was inconsistent. Most dietary, environmental and perinatal exposures relate to susceptibility, not course.

**Conclusions:**

Current evidence supports candidate predictors, not a validated instrument, and inference is further complicated by confounding by indication. A multidimensional, externally validated POMS-specific prognostic score remains to be developed—the aim of the ongoing PROMISING study.

**Supplementary Information:**

The online version contains supplementary material available at 10.1007/s00415-026-14029-z.

## Introduction

Multiple sclerosis (MS) is the most common non-traumatic cause of neurological disability in young adults [1]. Paediatric-onset MS (POMS), defined by disease onset before 18 years of age, is comparatively rare, accounting for 3–10% of all MS cases, with a pooled incidence of 0.87 per 100,000 and an estimated global prevalence of 2.53/100,000 (adjusted prevalence 1.48/100,000) [2, 3]. Children under 10 years account for 17–30% of paediatric cases, and the female-to-male ratio rises with age, from 0.8:1 below 6 years to 2.1:1 above 10 years, approaching the adult ratio of approximately 3:1 [4–6].

POMS is characterised by a high early relapse rate and a markedly inflammatory early course [7]. Although children recover from individual relapses better than adults, they reach disability milestones and secondary progression at a younger chronological age, and cognitive impairment may emerge in the first years of disease, with lasting consequences for education and socio-professional attainment [8–10]. The clinical priority is therefore to identify, at or shortly after diagnosis, the children whose disease will follow an aggressive trajectory, so that treatment can be escalated before irreversible damage accrues.

Predicting individual disease evolution remains unsolved in both adult and paediatric MS. Composite prognostic indices have been proposed in adult-onset MS and in radiologically isolated syndrome [11–13], but data in POMS are scarce, and real-world registry data may offer generalisable insights in this population [14]. The present review is conducted within the framework of the PROMISING study (Next Generation EU – NRRP M6C2 – Investment 2.1, PNRR-MAD-2022–12376868), which aims to develop a POMS-specific prognostic score integrating clinical, radiological, biological, psychosocial and environmental variables.

A recurrent difficulty in this literature is conceptual rather than empirical. Studies described as addressing “prognostic factors” in POMS in fact address at least three distinct questions, with different source populations and different time-zero points. Conflating them undermines the construction of a prognostic model, because variables that predict who develops MS carry no information about how an already-diagnosed child will fare. We therefore begin by making this distinction explicit, and we apply it consistently throughout the review.

### A conceptual framework: susceptibility, conversion and prognosis

We distinguish three axes of inference (Table 1).

**Table 1:** Three axes of inference in the paediatric MS literature

Axis	Question addressed	Source population/time zero	Typical outcomes	Representative factors in POMS
Susceptibility	Who develops POMS?	General paediatric population; time zero = exposure window	Disease occurrence; age at onset	BMI/obesity; vitamin D; passive smoke; EBV/HSV-1; breastfeeding; caesarean delivery; maternal illness in gestation; parental occupational exposure; air pollution; household pesticides
Conversion (diagnostic prediction)	Which children with CIS/ADS will develop MS?	Children with a first demyelinating event; time zero = first event	Second clinical attack; fulfilment of MS criteria	MS-like baseline MRI; CSF OCBs; optic nerve involvement; multifocal onset; older age at presentation; female sex
Prognosis (post-diagnosis)	Among children with established POMS, who will do worse?	Children with confirmed POMS; time zero = diagnosis	Relapse activity; EDSS worsening; time to EDSS 4.0/6.0; SPMS conversion; cognitive decline; educational/vocational attainment; QoL	Early relapse number and inter-attack interval; ARR; early EDSS change; brainstem/spinal cord lesions; T2 lesion accrual; thalamic and NAWM metrics; sNfL; DMT delay; early HET exposure

*ADS* acquired demyelinating syndrome, *ARR* annualised relapse rate, *CIS* clinically isolated syndrome, *CSF* cerebrospinal fluid, *DMT* disease-modifying therapy, *EBV* Epstein–Barr virus, *EDSS* Expanded Disability Status Scale, *HET* high-efficacy therapy, *HSV-1* herpes simplex virus type 1, *NAWM* normal-appearing white matter, *OCB* oligoclonal band, *QoL* quality of life, *sNfL* serum neurofilament light chain, *SPMS* secondary progressive multiple sclerosis

*Susceptibility factors* answer the question: who develops POMS? The source population is the general paediatric population, time zero is the exposure window (often prenatal or early childhood), and the outcome is disease occurrence or age at onset. Obesity, vitamin D status, passive smoke exposure, Epstein–Barr virus (EBV) seropositivity, breastfeeding, mode of delivery and air pollution belong primarily to this axis.

*Conversion (diagnostic) predictors* answer the question: which children presenting with a clinically isolated syndrome (CIS) or an acquired demyelinating syndrome (ADS) will develop MS? The source population comprises children with a first demyelinating event who do not yet have an MS diagnosis; time zero is that event; the outcomes are a second clinical attack or fulfilment of MS diagnostic criteria. MS-like baseline MRI, cerebrospinal fluid (CSF) oligoclonal bands (OCBs), optic nerve involvement, multifocal onset and older age at presentation belong to this axis.

*Prognostic factors* answer the question: among children with established POMS, who will do worse? The source population is restricted to children with confirmed POMS; time zero is diagnosis (or first event in patients subsequently confirmed as MS); the outcomes are relapse activity, disability accrual (EDSS worsening, time to EDSS 4.0/6.0), conversion to secondary progressive MS (SPMS), cognitive decline and, increasingly, educational and vocational attainment and quality of life.

These axes are not mutually exclusive: a small number of factors—notably vitamin D status and body mass index (BMI)—have been examined both as susceptibility factors and, separately, as modifiers of relapse rate after diagnosis. Where this is the case, we report the two bodies of evidence separately rather than merging them. Studies of paediatric CIS/ADS are retained in this review because they inform the earliest clinical decision points in POMS care, but they are presented in a dedicated section and are not treated as evidence of prognosis in confirmed POMS.

## Methods

This is a narrative review, reported in line with the SANRA guidance for narrative review articles [15]. It is not a systematic review, and no meta-analysis was performed.

### Search strategy

PubMed was searched from 1 January 2002 to 31 December 2025, with additional hand searching of pivotal paediatric cohorts published up to early 2026 during manuscript revision. The following string was used, combining MeSH terms and free-text (tiab) descriptors:

("Multiple Sclerosis"[Mesh] OR "multiple sclerosis"[tiab]) AND ("Child"[Mesh] OR "Adolescent"[Mesh] OR pediatric*[tiab] OR paediatric*[tiab] OR child*[tiab] OR adolescen*[tiab] OR juvenile[tiab] OR "childhood-onset"[tiab] OR "early-onset"[tiab]) AND ("Prognosis"[Mesh] OR "Disease Progression"[Mesh] OR "Risk Factors"[Mesh] OR prognos*[tiab] OR predict*[tiab] OR "disability progression"[tiab] OR "relapse rate"[tiab] OR "secondary progressive"[tiab] OR outcome*[tiab])

A structured literature search was performed in PubMed and Google Scholar covering the period 2002–2025, and re-run in PubMed on 15 July 2026 to confirm completeness. Using the search string reported above, restricted to human studies published in English, the PubMed query returned 3385 records. Titles and abstracts were screened for relevance to paediatric-onset MS and to at least one prognostic, conversion or susceptibility outcome. Google Scholar was searched as a supplementary source, using the same conceptual blocks in simplified free-text form, to capture recent or not-yet-indexed literature; records were screened by relevance until no further eligible studies emerged. Approximately 50 full-text articles were assessed and considered for inclusion in this narrative synthesis.

### Eligibility criteria

*Population.* Patients with MS onset before 18 years of age, diagnosed according to the criteria in force at the time of the study (2017 McDonald criteria for the most recent cohorts; IPMSSG definitions [16]). Studies of paediatric CIS/ADS were eligible but were analysed separately, as predictors of conversion rather than of prognosis (see Table 1).

*Exposures.* Demographic, clinical, radiological, laboratory, treatment-related, dietary/environmental and perinatal/parental factors, both modifiable and non-modifiable.

*Outcomes.* Disease activity (relapse rate; new T2 or gadolinium-enhancing lesions), disability progression (EDSS change; time to EDSS 4.0/6.0; SPMS conversion), cognitive outcomes, educational and socio-professional attainment, quality of life and biomarker trajectories.

*Study designs.* Observational (prospective, retrospective, case–control, cross-sectional), registry-based analyses, post hoc analyses of randomised trials and Mendelian randomisation studies. Priority was given to real-world cohort studies.

*Exclusions.* Case reports and case series with fewer than 10 patients; studies of adult-onset MS without a paediatric subgroup; studies investigating genetic susceptibility only, without any clinical prognostic analysis; articles not available in English.

### Classification and grading of the evidence

Each factor was first assigned to one of the three axes of Table 1. Factors relating to prognosis after POMS diagnosis were then graded into four tiers, applied by consensus among the authors:

*Tier A—Replicated.* Consistent direction of association across at least two independent cohorts, with at least one multivariable-adjusted analysis.

*Tier B—Promising but preliminary.* Biologically plausible association reported in a single cohort, or in small or unreplicated studies.

*Tier C—Susceptibility rather than prognosis.* Evidence pertains to the risk of developing POMS (or to conversion from CIS), without demonstrated effect on post-diagnosis course.

*Tier D—Inconsistent.* Conflicting directions of association across cohorts.

This grading is a structured, transparent appraisal of consistency and replication; it is not a formal risk-of-bias assessment. Established instruments exist for that purpose—QUIPS for prognostic factor studies [17] and PROBAST for prediction model studies [18]—and their application would require a systematic, dual-reviewer process beyond the scope of a narrative review. We regard this as a limitation (see Limitations) and note that a formal appraisal will be undertaken for the systematic evidence synthesis underpinning the PROMISING score.

When available, study-specific effect estimates (risk ratio, odds ratio, hazard ratio, incidence rate ratio) with 95% confidence intervals were extracted. Where multiple control groups were used, healthy controls were selected.

## Results

Results are presented by axis of inference and, within each domain, by the outcome the factor predicts. A structured summary by domain and outcome is provided in Supplementary Tables S1–S5; the evidence tier assigned to each candidate prognostic factor is given in Table 2.

**Table 2:** Evidence tiers for candidate prognostic factors in POMS, with the outcome each factor predicts

Factor	Outcome predicted	Tier	Rationale/caveat
Relapse number (first 2 years); short inter-attack interval; ARR	Time to EDSS 3.0/4.0/6.0; SPMS conversion	A—Replicated	Consistent across ≥ 4 independent cohorts, multivariable-adjusted [19–21]
EDSS change in first 2 years	9-year EDSS; disability worsening	A—Replicated	Large effect sizes; distinct from baseline EDSS [22]
Brainstem and spinal cord lesion topography; ≥ 2 new T2 lesions	9-year EDSS; relapse rate	A—Replicated	Concordant with adult MS; single large POMS cohort plus CIS data [22, 23]
Serum NfL	MRI/clinical activity; treatment escalation	A—Replicated	Multiple designs incl. RCT post hoc [24–27]; needs paediatric age-adjusted Z-scores and assay harmonisation
DMT delay; early high-efficacy therapy	Sustained EDSS 4.0; time to first relapse; disability worsening	A—Replicated	Registry-based [28–30]; subject to confounding by indication
Thalamic volume; NAWM integrity; infratentorial lesion number	Time to first relapse; 6-month CDW	B—Preliminary	Single 12-year cohort, *n* = 52 [31]; needs paediatric normative data and external validation
Serum GFAP; CHI3L1; CSF kappa free light chains	Relapse activity; progression	B—Preliminary	Strong adult rationale [32–35]; minimal paediatric data [36]
Cognitive impairment at onset; cognitive reserve	Cognitive trajectory; socio-professional attainment	B—Preliminary	Single 12-year cohort, *n* = 48 [10]; reserve rarely measured [37]
Sleep quality; psychiatric comorbidity; social determinants	Fatigue; cognition; MRI outcomes	B—Preliminary	Cross-sectional or associative [38–42]
Dietary fat/vegetable intake; physical activity; vitamin D status after diagnosis	Relapse rate	B—Preliminary	Post-diagnosis outcomes but self-reported exposure, single cohorts [43–46]
BMI/obesity; breastfeeding; caesarean delivery; maternal illness; parental occupational exposure; air pollution; household pesticides; EBV/HSV-1; passive smoke	Risk of developing POMS; age at onset	C—Susceptibility	Not shown to predict post-diagnosis course [47–56]; relevant to prevention, not prognostic modelling
Baseline EDSS	Long-term EDSS vs confirmed improvement	D—Inconsistent	Opposite directions across cohorts [22, 28]; plausibly regression to the mean and unstandardised assessment timing
Gadolinium-enhancing brainstem/cervical lesions at year 1	9-year EDSS	D—Inconsistent	Negative coefficient unexplained [22]; likely detection timing and treatment response rather than protective inflammation
Absence of mental-state involvement at onset	Third attack; EDSS > 4	D—Inconsistent	Plausibly diagnostic misclassification (ADEM) in older cohorts [20, 16, 57]

*ADEM* acute disseminated encephalomyelitis, *ARR* annualised relapse rate, *CDW* confirmed disability worsening, *CHI3L1* chitinase-3-like protein 1, *CIS* clinically isolated syndrome, *DMT* disease-modifying therapy, *EDSS* Expanded Disability Status Scale, *GFAP* glial fibrillary acidic protein, *NAWM* normal-appearing white matter, *NfL* neurofilament light chain, *POMS* paediatric-onset multiple sclerosis, *SPMS* secondary progressive multiple sclerosis

### Predictors of conversion from CIS/ADS to MS

Studies of children presenting with a first demyelinating event address the diagnostic rather than the prognostic question, and are summarised here separately. In a cohort of 770 children with paediatric CIS, female sex, multifocal onset and optic nerve involvement were independently associated with a second clinical attack [9]. In the KIDMUS cohort of 296 children with a first CNS demyelinating event, an MS-like baseline MRI and optic nerve lesions predicted a second attack, and corpus callosum perpendicular lesions were associated with increased risk of a second demyelinating event [23]. The presence of CSF OCBs at first presentation was associated with conversion to MS and with a more severe subsequent course [23, 58]. In a prospective national cohort of children with acute demyelination, at least one brain lesion on baseline MRI (HR 37.9) and CSF OCBs (HR 6.33) were the strongest determinants of MS diagnosis [58]. In a recent French paediatric cohort, multifocal onset and optic nerve involvement were again associated with progression to MS [19].

These variables are clinically relevant at the point of first presentation, but they characterise the transition into MS. Their prognostic value after a POMS diagnosis has been confirmed has, for most of them, not been established, and they are therefore not carried forward as prognostic factors.

### Prognostic factors after POMS diagnosis

#### Demographic factors

Age at onset has been examined against three different outcomes, and the apparent inconsistency in the literature largely dissolves once these are separated.

*Relapse recovery.* In a longitudinal study of 132 POMS patients, younger age was associated with better recovery of EDSS after a relapse: for every 10 years of age, EDSS recovery was reduced by 0.15 points (*p* < 0.001) [8].

*First EDSS worsening.* In the paediatric CIS cohort, onset before 15 years was associated with a lower risk of a first EDSS-worsening event (HR 0.59, 95% CI 0.42–0.83) [9].

*Timing of relapse activity.* In a French cohort, children older than 11.5 years relapsed earlier and had a worse prognosis [19]; in a Swedish registry cohort, onset at or after 14 years was associated with a shorter time to sustained disability milestones [21].

Taken together, younger children appear to recover better from individual relapses, while older children accrue relapse activity earlier. These are complementary rather than contradictory observations, but they must be kept distinct when age is entered into a prognostic model, since the direction of the association depends on the outcome modelled. Female sex has been associated with a second clinical attack in paediatric CIS [9] and belongs primarily to the conversion axis; its independent prognostic value after POMS diagnosis is not established.

#### Clinical factors

*Early inflammatory activity—relapse-related variables.* These are the strongest and most reproducible prognostic factors identified. A higher relapse number in the first two years predicted a shorter time to SPMS (HR 4.52, *p* = 0.016) and to disability milestones [21]. A first inter-attack interval shorter than one year predicted a higher risk of a third demyelinating attack or EDSS > 4 (HR 1.56, 95% CI 1.07–2.28) [20] and a higher hazard of SPMS conversion [57, 59]. A higher annualised relapse rate (ARR) in the first five years predicted shorter time to EDSS 3.0 (HR 4.62, 95% CI 3.40–6.28), EDSS 4.0 (HR 4.64, 95% CI 3.02–7.15) and EDSS 6.0 (HR 2.69, 95% CI 1.42–5.11) [59]. Conversely, complete remission after the first relapse was associated with a lower disability risk (EDSS 3.0: HR 0.42, 95% CI 0.21–0.83) [21], and a longer first remission predicted a longer time to secondary progression [19, 59]. The severity of, and incomplete recovery from, the first relapse was associated with a higher hazard of reaching disability milestones [21]. These associations are consistent across cohorts spanning two decades and multiple health systems (Tier A).

*Early EDSS change.* EDSS change within the first two years after onset strongly predicted long-term disability (9-year disability worsening: OR 13.40 for 1-year EDSS change, *p* < 0.001; OR 16.38 for 2-year change, *p* = 0.02) [22]. Early EDSS *change* should be distinguished from *baseline* EDSS, discussed below.

*Baseline EDSS: an inconsistent predictor.* Two cohorts point in opposite directions. In 123 POMS patients, higher baseline EDSS predicted higher 9-year EDSS (*β* = 0.58, *p* < 0.001) [22]. In 291 POMS patients from the Danish registry, baseline EDSS ≥ 3 was associated with an approximately two-fold higher likelihood of confirmed clinical improvement [28]. Rather than a genuine paradox, this most plausibly reflects regression to the mean and outcome definition: a patient assessed shortly after a severe relapse has a high EDSS that is expected to fall as the relapse resolves, so a high baseline EDSS predicts improvement when improvement is the outcome and worse disability when long-term EDSS is the outcome. The timing of the baseline assessment relative to the last relapse is rarely reported and is a plausible source of heterogeneity. Baseline EDSS as measured in these cohorts is therefore an unreliable candidate for a prognostic score unless the assessment window is standardised (Tier D).

*Functional system involvement.* Absence of mental-state involvement at onset was associated with higher disease severity (HR 1.91, 95% CI 1.05–3.49) and supratentorial onset symptoms appeared protective against subsequent disability worsening [20, 57 ]. This counter-intuitive finding may reflect diagnostic misclassification: encephalopathic presentations are more frequently associated with acute disseminated encephalomyelitis than with MS in children [16], so cohorts assembled before the current criteria may have included ADEM cases with a more favourable course. This association should be interpreted with caution and is not, in our view, a robust candidate predictor.

*Disease course and duration.* Progressive onset, although rare in POMS, predicted increased disability and shorter time to EDSS 4.0 [21]. Disease duration ≥ 2 years was associated with a 62–84% reduction in the chance of EDSS improvement and with higher relapse rates [28].

#### Radiological factors

*Conventional MRI: lesion topography and burden.* Lesion location carries distinct prognostic implications. Brainstem lesions predicted higher 9-year EDSS (*β* = 0.31, *p* = 0.04) and cervical cord lesions predicted both higher 9-year EDSS (*β* = 0.22, *p* = 0.05) and higher relapse rates (*β* = 0.16, *p* = 0.003); optic nerve lesions predicted a shorter time to first relapse (HR 2.10, *p* = 0.02); cerebellar lesions were associated with a lower relapse rate (*β* = − 0.15, *p* < 0.001) [22]. Meeting MS diagnostic criteria at first MRI predicted higher disease severity (HR 1.89, 95% CI 1.26–2.85) [20]. On follow-up imaging, ≥ 2 new T2 lesions within two years predicted 9-year disability worsening (OR 4.91, *p* = 0.02) [22].

*An apparent paradox.* In the same cohort, gadolinium-enhancing brainstem and cervical cord lesions at year 1 were associated with *lower* 9-year EDSS (cervical cord *β* = − 0.41, *p* = 0.02; brain *β* = − 0.29, *p* = 0.05) [22]. We previously described this as suggesting a protective effect of inflammation. On reflection this interpretation is too strong. Gadolinium enhancement is a transient phenomenon whose detection depends on the timing of imaging relative to relapse; enhancing lesions identify patients scanned close to an acute, potentially treatment-responsive inflammatory event, who may subsequently be escalated to more effective therapy. The negative coefficient is therefore at least as compatible with detection timing and treatment response as with a biologically protective effect of inflammation, and we now present it as an unexplained finding requiring replication (Tier D).

*Beyond lesion counts: atrophy, regional volumes and network integrity.* Prognostic MRI research in MS is moving beyond lesion-centric metrics, and this shift is particularly relevant in children, whose brains are still developing: POMS is associated with reduced brain volume at first presentation and with subsequent failure of age-expected brain growth, rather than with atrophy from a normal baseline alone [60]. In a 12-year multicentre study of 52 POMS patients, infratentorial lesion number (HR 1.09, *p* = 0.016), spinal cord lesion presence (HR 2.45, *p* = 0.017) and thalamic volume (HR 0.77, *p* = 0.046) were associated with a shorter time to first relapse; white matter lesion volume predicted recurrent relapses (HR 1.04, *p* = 0.013); and lower fractional anisotropy of normal-appearing white matter predicted a shorter time to 6-month confirmed disability worsening (HR 0.67, *p* = 0.004) and EDSS worsening (*β* = − 0.26, *p* = 0.029) [31]. Grey matter and thalamic damage have been linked to cognitive impairment in paediatric MS [61], and functional network reorganisation has been proposed as a substrate of cognitive reserve [62]. These metrics are biologically compelling but rest, in POMS, on single cohorts of modest size; their incorporation into a prognostic score will require normative paediatric reference values, harmonised acquisition protocols and external validation (Tier B).

#### Fluid biomarkers

*Serum neurofilament light chain.* sNfL is the fluid biomarker with the strongest evidence in POMS. Higher sNfL levels were associated with greater cerebral and spinal lesion burden, higher EDSS, higher relapse frequency and recent clinical or radiological activity within 90 days [24–26]. Untreated paediatric MS patients had markedly higher levels than controls (median 19.0 vs 4.6 pg/mL, *p* < 0.001), levels rose by 51.1% after a recent relapse and by 9.1% per contrast-enhancing lesion, and higher sNfL was associated with subsequent escalation to high-efficacy therapy (OR 2.60, *p* = 0.024) [25]. In a post hoc analysis of the TERIKIDS trial, each doubling of baseline plasma NfL increased the hazard of subsequent radiological or clinical activity [27]. sNfL also declined after initiation of disease-modifying therapy (DMT) [25], which confirms its responsiveness but, as discussed below, complicates its use as a baseline prognostic marker in treated cohorts (Tier A).

*Paediatric-specific measurement issues.* Three caveats apply specifically to children. First, sNfL concentrations vary with age and body mass index, so raw values are difficult to interpret across the paediatric age range; age-adjusted *Z*-scores derived from normative databases are now the preferred metric in adults [63] and equivalent paediatric reference values are needed. Second, assay platforms (Simoa, Ella) are not fully interchangeable, and multicentre studies require cross-calibration. Third, several paediatric sNfL cohorts comprise mixed acquired demyelinating syndromes, including MOG antibody-associated disease, in which sNfL is also elevated [24]; estimates derived from such cohorts may not transfer directly to confirmed POMS.

*Serum glial fibrillary acidic protein.* sGFAP, released upon astroglial activation, has emerged in adult MS as a marker with a profile distinct from sNfL: it is more closely associated with progression independent of relapse activity and with chronic inflammatory burden, whereas sNfL tracks acute neuroaxonal injury [32, 33]. Paediatric data are scarce but suggest that sGFAP is measurable and informative in POMS and may complement sNfL [36]. Whether the sNfL/sGFAP combination improves prognostic discrimination in children over sNfL alone is, to our knowledge, untested (Tier B).

*Other candidate biomarkers.* CSF OCBs at diagnosis have been linked to a more severe course [23], although much of the supporting evidence derives from CIS cohorts and thus pertains chiefly to conversion. Chitinase-3-like protein 1 (CHI3L1), a marker of astroglial and microglial activation, predicts conversion from CIS to MS and disability accrual in adults [35], with limited paediatric data. CSF kappa free light chains have been proposed as a robust and less operator-dependent alternative to OCBs [34]. Each of these remains preliminary in POMS (Tier B).

#### Treatment-related factors and treatment as a confounder

*Timing and class of therapy.* In the Danish registry, a two-year delay in DMT initiation increased the risk of reaching sustained EDSS 4.0 by 2.5-fold (HR 2.52, 95% CI 1.01–6.34), each additional year of delay raised that risk by 17% (HR 1.17, 95% CI 1.05–1.30) and reduced the probability of confirmed EDSS improvement by 10% (HR 0.90, 95% CI 0.84–0.96) [28]. Early exposure to high-efficacy therapies (HETs) was associated with a longer time to first relapse (HR 0.20, *p* = 0.010) and a lower relapse rate [22, 31]. Registry analyses support this directly in POMS: in the Italian MS Registry, HETs reduced the hazard of disability worsening across disability states (HR 0.41, 95% CI 0.31–0.53 in the minimal-disability state) [29]; a longitudinal analysis of global and national registries reached concordant conclusions [30]; and in a French national cohort, first-line HETs reduced the risk of a first relapse by 54% (HR 0.46, 95% CI 0.31–0.67, *p* < 0.001) [64]. These four studies [29, 30, 64, 65] constitute the direct evidence base for early high-efficacy treatment in POMS; the fingolimod PARADIGMS trial provides the only randomised comparison of a HET against an injectable in this population.

*Confounding by indication.* Treatment is not a background nuisance in this literature but a systematic source of bias, and it operates in a direction that attenuates the very associations reviewed here. Children with a more aggressive presentation—high relapse rate, heavy lesion burden, high sNfL—are precisely those escalated earliest to high-efficacy therapy. Their subsequent outcomes therefore reflect both their intrinsic disease severity and the more effective treatment they received. The consequence is bidirectional: the prognostic effect of adverse baseline features is likely *underestimated* in treated cohorts, while the observational benefit of HET is likely *confounded* by the fact that treated patients differ systematically from untreated ones. The problem is compounded for dynamic markers: sNfL, lesion accrual and relapse rate all change after treatment initiation, so a “baseline” measurement taken after DMT start is not a measure of natural history. Most of the studies reviewed here adjust for DMT exposure as a covariate, but few use methods designed for time-varying treatment (marginal structural models, target trial emulation [66]), and none, to our knowledge, has been applied to POMS. This is a central methodological obstacle to prognostic modelling in this population and, in our view, the single most important limitation of the evidence summarised in this review.

### Outcomes beyond EDSS: cognition, education and quality of life

The literature reviewed here—and consequently this review—is heavily EDSS centred. We acknowledge this explicitly, because in a disease beginning during brain development and schooling, the EDSS is arguably the least appropriate outcome available. POMS patients spend long periods at the lower end of the scale, where it is insensitive to change, while accumulating deficits that the scale does not measure at all.

*Cognitive trajectories.* Cognitive impairment affects roughly a third of children with MS early in the disease, with processing speed, sustained attention, memory and executive function most affected [67]. In a 12-year multicentre follow-up of 48 POMS patients, the prevalence of cognitive impairment rose from 21.2% at baseline to 54.5%; a higher pre-baseline relapse number predicted a worse cognitive trajectory (*β* = − 0.1, *p* = 0.025), while higher baseline IQ was protective (*β* = + 0.3, 95% CI 0.1–0.5, *p* = 0.017) [10]. Cognitive impairment at onset is thus both an outcome and a prognostic factor.

*Educational and socio-professional attainment.* In the same cohort, cognitively impaired patients had significantly worse socio-professional outcomes (*β* = 4.8, 95% CI 1.4–8.2, *p* = 0.008) [10]. For a disease with onset at a median age of 14 years, attained education and employment are arguably the outcomes that matter most to patients and families, and they are almost entirely absent from prognostic studies.

*Cognitive reserve.* Higher baseline IQ [10] and, in adults, larger maximal lifetime brain volume and greater intellectual enrichment [37] attenuate the effect of a given lesion burden on cognitive performance. Cognitive reserve is therefore a plausible effect modifier in POMS: two children with identical MRI burden may follow divergent cognitive trajectories. It is rarely measured and never, to our knowledge, incorporated into a paediatric prognostic model.

*Fatigue, sleep, psychiatric comorbidity and social determinants.* Poor sleep quality correlates with fatigue (*r* = 0.70, *p* < 0.01) and with EDSS (*r* = 0.36, *p* = 0.01) [38, 39]. Depression and anxiety are common and interact with fatigue and cognition [41]. Social determinants of health are associated with brain MRI outcomes in POMS [42], and social hardship is prevalent in this population and associated with worse outcomes [40]. These dimensions are prognostically relevant, potentially modifiable and systematically under-measured (Tier B).

A prognostic instrument for POMS that predicts EDSS alone would be answering a question that is not the one families ask. The PROMISING score will therefore include cognitive and psychosocial outcomes alongside disability.

### Susceptibility and environmental factors: context, not prognosis

The exposures summarised in this section are established or candidate determinants of the risk of developing POMS. With the partial exceptions noted below, they have not been shown to predict relapse activity, disability accrual, cognitive decline or SPMS conversion among children who already have a diagnosis of MS. They are presented here because they define the biological and epidemiological context of POMS and are targets for primary prevention, not because they are prognostic factors; accordingly, they are classified as Tier C and are not proposed as candidate variables for the prognostic score.

*Body mass index and obesity.* High BMI and obesity in childhood and adolescence are associated with a greater risk of POMS and an earlier age at onset [47–53]. Mendelian randomisation analysis supports a causal contribution of high BMI and low vitamin D to POMS risk [48]—a design worth emphasising, since it strengthens causal inference beyond conventional observational data. Evidence that BMI modifies the course of established POMS is limited.

*Vitamin D.* Vitamin D status straddles the two axes. Low vitamin D is associated with an increased risk of POMS [48] (susceptibility), and, separately, lower 25(OH)D levels have been associated with a higher relapse rate among children with established POMS [46], with vitamin D pathway genes influencing relapses [45]. The latter observations pertain to prognosis and are graded Tier B; the causal question is unresolved and interventional data are lacking. We have accordingly amended the earlier statement that vitamin D deficiency “predicted” worse outcomes to “was associated with” higher relapse rates.

*Diet and physical activity.* In a US multicentre study of 219 children with POMS or paediatric CIS, each 10% increase in energy intake from fat was associated with a 56% higher relapse risk, each 10% increase in saturated fat with a threefold higher risk and each additional cup-equivalent of vegetable intake with a 50% lower relapse risk [43]. These are among the few environmental associations measured against a post-diagnosis outcome (relapse rate) and are therefore genuinely prognostic in nature, though based on self-reported intake in a single cohort (Tier B). Lower physical activity is associated with higher disease burden [44]. We note that our earlier reference to iron intake was not supported by the cited dietary studies, which concern fat, saturated fat and vegetable intake; the claim has been removed.

*Tobacco smoke, infections and pollutants.* Passive smoke exposure increases MS risk, particularly in HLA-DRB1*15-positive children [54, 68, 69]; the PEDIGREE study frames reduction of early-life smoke exposure as a preventive strategy [54]. EBV and HSV-1 seropositivity are associated with higher disease risk [70]. Air pollutants and household pesticide exposure are associated with increased odds of POMS [71, 55]. All of these pertain to susceptibility.

*Perinatal and parental factors.* Lack of breastfeeding is associated with a higher risk of POMS (OR 1.68–11.71, *p* = 0.003) [72]. Maternal illness during gestation approximately doubled the odds of POMS, and paternal occupational exposure (gardening, pesticide use) was associated with increased risk [73, 58]. Caesarean delivery was reported as associated with a lower risk of POMS [73]; we previously described this as “protective”, which overstates observational evidence subject to confounding by indication for delivery, and we have revised the wording accordingly. None of these exposures has been shown to influence the course of established POMS.

*Gut microbiota.* Alterations in gut microbiota composition have been associated with immune activation and MS activity in children [74, 75]. The evidence remains exploratory and largely cross-sectional, and we have softened the earlier claim of a consistent correlation with disease activity accordingly.

## Discussion

Three conclusions follow from this synthesis. First, the prognostic signal in POMS is concentrated in a small number of variables, all of which index early inflammatory activity: relapse number and spacing in the first two years, early EDSS change, lesion topography and accrual, and sNfL. These are replicated, measurable in routine care and available in existing registries—the practical criteria for inclusion in a prognostic model. Second, a considerable proportion of what is described in the literature as “prognostic” in POMS is in fact evidence about who develops the disease, and cannot inform the management of a child who already has it. Third, the associations that do concern prognosis are systematically distorted by treatment, in ways that current analyses do not adequately address.

### From candidate predictors to a prognostic model

The step from a list of associations to a usable prognostic instrument is not merely additive, and the literature reviewed here does not accomplish it. Several methodological requirements deserve emphasis.

*Predictor selection.* Candidate predictors should be specified a priori on the basis of prior evidence and clinical availability, not selected by univariable screening of the development dataset, which inflates optimism and produces unstable models. The tiering in Table 2 is intended as a starting point for such a pre-specification.

*Sample size and events per variable.* POMS is rare, and the outcome events of interest (EDSS 6.0, SPMS conversion) are few and late. Formal sample-size calculations for prediction models [76] indicate that the number of candidate predictors that can be supported by a POMS cohort of realistic size is small—an argument for parsimonious models built on Tier A variables, and for pooling across registries, as PROMISING intends.

*Outcome definition and time horizon.* A score must predict a specified outcome over a specified horizon. “Progression” is not one outcome: relapse activity at 2 years, disability at 10 years and cognitive decline at 12 years require different models, and a variable useful for one may be useless for another—as the age-at-onset and baseline-EDSS examples above illustrate.

*Handling of treatment.* Because treatment is assigned on the basis of prognosis, models developed in treated cohorts estimate outcomes under prevailing treatment practice, not natural history. This is acceptable—and arguably more clinically useful—provided it is stated explicitly and the model is not interpreted causally. Where the aim is to estimate the effect of treatment strategies, methods for time-varying confounding (marginal structural models, target trial emulation [66]) are required.

*Validation and reporting.* Apparent performance in the development dataset is uninformative. Internal validation (bootstrapping), assessment of both discrimination and calibration, and external validation in an independent cohort are essential [77], and reporting should follow the TRIPOD statement [78]. No POMS prognostic score currently meets these standards.

It follows that the present review—and the literature it summarises—supports a set of candidate predictors, not a ready-to-use prognostic tool. We state this deliberately, since the framing of this review within the PROMISING study might otherwise be read as implying that the available evidence is already sufficient to define a validated score. It is not.

### Strengths and limitations

The main strengths of this review are its explicit separation of susceptibility, conversion and prognosis; its organisation of findings by the outcome predicted; its structured grading of the evidence; and its emphasis on real-world cohort data, which enhances applicability to routine practice.

Several limitations should be acknowledged. This is a narrative and not a systematic review: study selection, although guided by predefined criteria and a reported search strategy, was not conducted in duplicate, and no formal risk-of-bias assessment (QUIPS [17]) or prediction model appraisal (PROBAST [18]) was undertaken. The evidence tiering in Table 2 reflects consistency and replication, not internal validity, and was assigned by author consensus rather than by an independent, blinded process. The search was restricted to two databases and to English-language publications, which may have led to incomplete retrieval. The long inclusion window (2002–2026) spans successive revisions of the diagnostic criteria and a transformation of the therapeutic landscape, so cohorts assembled in the early 2000s—before the routine exclusion of MOGAD and NMOSD, and before high-efficacy therapies—are not directly comparable with contemporary ones; several of the counter-intuitive findings discussed above may be artefacts of this era effect. Many included studies were retrospective, single-centre and small, and prognostic factors were frequently assessed in isolation and without adequate adjustment for treatment exposure. Finally, most studies report EDSS-based outcomes; cognitive, educational and psychosocial outcomes, which are arguably the most consequential in this population, are comparatively neglected, and this review inevitably reflects that imbalance.

## Conclusions

Prognostic determinants in POMS span demographic, clinical, radiological, biological, treatment-related and environmental domains, but these domains do not contribute equally, and not all of them contribute to prognosis at all. The most reproducible predictors of long-term outcome after a POMS diagnosis are indicators of early inflammatory activity—relapse number and spacing in the first two years, early EDSS change, annualised relapse rate—together with lesion topography and accrual on MRI and serum neurofilament light chain. Advanced MRI metrics, serum GFAP and other fluid biomarkers, and measures of cognitive reserve and psychosocial context are biologically promising but remain preliminary. Most dietary, environmental and perinatal exposures relate to the risk of developing POMS rather than to its course, and should not be treated as prognostic variables.

In current practice, the available evidence supports early initiation of high-efficacy therapy in children presenting with adverse prognostic features, structured monitoring with serial MRI and fluid biomarkers, and attention to modifiable factors and to cognitive and psychosocial functioning. It does not yet support a validated, multidimensional prognostic score: such a tool must still be developed on pre-specified candidate predictors, in cohorts large enough to support it, with explicit handling of treatment as a time-varying confounder, and—crucially—externally validated before it can be used to guide individual treatment decisions. This is the objective of the ongoing PROMISING study.

## Supplementary Information

Below is the link to the electronic supplementary material.

Supplementary file1 (DOCX 2039 KB)

## Data Availability

Not applicable; no new datasets were generated or analysed.
