# Supplementary material for: Prognostic factors in paediatric-onset multiple sclerosis: a narrative review
Source: J Neurol. 2026 Jul 29;273(8):495. doi: 10.1007/s00415-026-14029-z (PMC13421221; doi:10.1007/s00415-026-14029-z)
Supplement: Supplementary file 1 — Supplementary file1 (DOCX 2039 KB) [file 415_2026_14029_MOESM1_ESM.docx]

**Supplementary Material**

**Prognostic Factors in Pediatric-Onset Multiple Sclerosis: A Narrative Review**

*Manuscript JOON-D-26-03298 — Journal of Neurology*

Supplementary Tables S1–S5 replace the single Table 1 of the original submission, and are organised by domain as requested. For every factor, the **axis of inference** column states whether the evidence pertains to susceptibility (risk of developing POMS), conversion (from CIS/ADS to MS) or prognosis (course after a POMS diagnosis); the **outcome predicted** column states the specific endpoint against which the factor was assessed; and the **evidence tier** column reports the grading defined in the Methods and summarised in Table 2 of the main text.

**Evidence tiers: A** = replicated (consistent direction across ≥2 independent cohorts, ≥1 multivariable-adjusted); **B** = promising but preliminary (single cohort, small or unreplicated); **C** = susceptibility rather than prognosis (pertains to disease risk or age at onset, not to post-diagnosis course); **D** = inconsistent (conflicting directions across cohorts).

**Table S1. Demographic and clinical factors**

| **Factor** | **Study [ref]** | **Design; N** | **Axis of inference** | **Outcome predicted** | **Effect estimate** | **Evidence tier** |
| --- | --- | --- | --- | --- | --- | --- |
| **Age at onset (younger)** | Chitnis 2020 [13] | Longitudinal prospective; 132 POMS | Prognosis | EDSS recovery 6 months after relapse | Per 10 years of age, EDSS recovery reduced by 0.15 points (p < 0.001); odds of EDSS not improving increased 1.33-fold (p < 0.0001) | A |
| **Age at onset < 15 years** | Iaffaldano 2017 [14] | Observational; 770 pediatric CIS | Prognosis | Time to first confirmed EDSS-worsening event | HR 0.59 (95% CI 0.42–0.83) | A |
| **Age at onset > 11.5 years** | de Chalus 2025 [15] | Multicentre retrospective (KidBioSEP); French 70 pediatric MS cohort | Prognosis | Time to relapse; severity of course | Earlier relapse and worse prognosis in children > 11.5 years (predictive model) | A |
| **Age at onset ≥ 14 years** | McKay 2019 [20] | Retrospective cohort; 549 POMS | Prognosis | Time to sustained EDSS 3.0/4.0/6.0 | HR 5.78 (univariable analysis) | B |
| **Female sex** | Iaffaldano 2017 [14] | Observational; 770 pediatric CIS | Conversion | Time to second clinical attack | Independent predictor of second clinical attack (multivariable Cox model) | — |
| **Baseline EDSS (higher)** | De Meo 2021 [16] | Longitudinal observational cohort; 123 POMS | Prognosis | 9-year EDSS score | β = 0.58, p < 0.001 (linear regression) | D |
| **Baseline EDSS ≥ 3** | Kopp 2020 [17] | Observational cohort; 291 POMS | Prognosis | 6-month confirmed EDSS improvement | ~2-fold higher likelihood of confirmed clinical improvement vs EDSS < 3 | D |
| **EDSS change in first 2 years** | De Meo 2021 [16] | Longitudinal observational cohort; 123 POMS | Prognosis | 9-year disability worsening; 9-year EDSS | 1-year EDSS change: OR 13.40, p < 0.001; 2-year change: OR 16.38, p = 0.02. 9-year EDSS: β = 0.71 and β = 0.55, p < 0.001 | A |
| **Relapse number in first 2 years** | McKay 2019 [20] | Retrospective cohort; 549 POMS | Prognosis | Time to secondary progression | HR 4.52, p = 0.016 (multivariable Cox) | A |
| **Relapse occurrence (any)** | Iaffaldano 2017 [14] | Observational; 770 pediatric CIS | Prognosis | Time to first EDSS-worsening event | HR 5.08 (95% CI 3.46–7.46) | A |
| **Annualised relapse rate (first 5 years)** | Boiko 2002 [21] | Prospective longitudinal; 116 POMS | Prognosis | Time to EDSS 3.0/4.0/6.0; SPMS conversion | EDSS 3.0: HR 4.62 (3.40–6.28); EDSS 4.0: HR 4.64 (3.02–7.15); EDSS 6.0: HR 2.69 (1.42–5.11). ARR > 0.6 predicted shorter time to SPMS | A |
| **Short inter-attack interval (1st–2nd attack ≤ 1 year)** | Mikaeloff 2006 [18] | Prospective; 197 POMS | Prognosis | Third demyelinating attack; EDSS > 4 | HR 1.56 (95% CI 1.07–2.28), p = 0.02 | A |
| **First inter-attack interval < 1 year** | McKay 2019 [20] | Retrospective cohort; 549 POMS | Prognosis | Time to disability milestones | HR 3.09–4.27, p ≤ 0.04 | A |
| **Severity of / incomplete recovery from first relapse** | McKay 2019 [20] | Retrospective cohort; 549 POMS | Prognosis | Time to EDSS 3.0/4.0/6.0; recurrence of relapses | Higher hazard of reaching disability milestones (multivariable Cox) | A |
| **Complete remission after first relapse** | Boiko 2002 [21]; McKay 2019 [20] | Prospective longitudinal (116 POMS); retrospective cohort (549 POMS) | Prognosis | Time to EDSS 3.0/4.0 | EDSS 3.0: HR 0.42 (0.21–0.83); EDSS 4.0: HR 0.41 (0.18–0.94) | A |
| **Longer first remission** | Boiko 2002 [21]; de Chalus 2025 [15] | Prospective longitudinal; multicentre retrospective | Prognosis | Time to SPMS conversion | Longer time between 1st and 2nd attack associated with lower hazard of SPMS | A |
| **Time to first relapse (shorter)** | De Meo 2021 [16] | Longitudinal observational cohort; 123 POMS | Prognosis | ARR at year 2 | β = −0.12, p = 0.01 (linear regression) | B |
| **Progressive / secondary progressive course** | McKay 2019 [20]; Simone 2002 [19] | Retrospective cohorts; 549 and 83 POMS | Prognosis | Time to EDSS 4.0; time to secondary progression | Secondary progressive course: HR 11.07, p < 0.001 [20]; progressive course: HR 2.93 (1.37–6.26), p = 0.01 [19] | A |
| **Disease duration ≥ 2 years** | Kopp 2020 [17] | Observational cohort; 291 POMS | Prognosis | Confirmed EDSS improvement; relapse rate | 62–84% reduction in chance of EDSS improvement; relapse rate ratio 1.50 (1.05–2.15), p < 0.05 | A |
| **Multifocal / polysymptomatic onset** | Iaffaldano 2017 [14]; de Chalus 2025 [15] | Observational (770 ped CIS); multicentre retrospective | Conversion | Second clinical attack; conversion to MS | Independently associated with second attack and with progression to MS (multivariable models) | — |
| **Optic nerve involvement at onset** | Iaffaldano 2017 [14]; de Chalus 2025 [15] | Observational (770 ped CIS); multicentre retrospective | Conversion | Second clinical attack; conversion to MS | Significantly associated with subsequent attack and conversion to definite MS | — |
| **Absence of mental-state involvement at onset** | Simone 2002 [19] | Retrospective cohort; 83 POMS | Prognosis | Third demyelinating attack; EDSS > 4 | HR 1.91 (95% CI 1.05–3.49), p = 0.04. Interpretation confounded by possible ADEM misclassification in pre-criteria cohorts | D |
| **Supratentorial onset symptoms** | Mikaeloff 2006 [18]; Simone 2002 [19] | Prospective (197 POMS); retrospective (83 POMS) | Prognosis | Subsequent disability worsening | Reported as protective against disability worsening; direction not replicated in later cohorts | D |
| **Cognitive impairment at onset** | Portaccio 2022 [22] | Multicentre longitudinal; 48 POMS (12-year follow-up) | Prognosis | Cognitive trajectory; socio-professional attainment (WSAS) | Cognitive impairment rose from 21.2% to 54.5% over 12 years. Higher baseline IQ protective (β = +0.3, 95% CI 0.1–0.5, p = 0.017); higher pre-baseline relapse number adverse (β = −0.1, p = 0.025). Worse socio-professional outcome in impaired patients (β = 4.8, 95% CI 1.4–8.2, p = 0.008) | B |
| **Poor sleep quality** | MacAllister 2009 [24] | Cross-sectional observational; 51 POMS | Prognosis | Fatigue; quality of life; EDSS correlation | Correlation with EDSS r = 0.36, p = 0.01; with fatigue r = 0.70 (self-reported) and r = 0.77 (parent-reported), p < 0.01 | B |
| **Psychosocial stressors (family dynamics; mental-health issues)** | MacAllister 2007 [25] | Narrative review; n.a. | Prognosis | Symptom burden; overall prognosis | Narrative synthesis; no quantitative effect measure | B |
| **Social determinants of health; social hardship** | Ross 2024 [60]; Wilson 2024 [61] | Observational cohorts (US Network of Pediatric MS Centers) | Prognosis | Brain MRI outcomes; access to care; disease outcomes | Social determinants of health associated with brain MRI outcomes [60]; social hardship prevalent and associated with worse outcomes [61] | B |

*ADEM, acute disseminated encephalomyelitis; ARR, annualised relapse rate; CI, confidence interval; CIS, clinically isolated syndrome; EDSS, Expanded Disability Status Scale; HR, hazard ratio; OR, odds ratio; POMS, pediatric-onset multiple sclerosis; SPMS, secondary progressive multiple sclerosis; WSAS, Work and Social Adjustment Scale.*

**Table S2. Radiological (MRI) factors**

| **Factor** | **Study [ref]** | **Design; N** | **Axis of inference** | **Outcome predicted** | **Effect estimate** | **Evidence tier** |
| --- | --- | --- | --- | --- | --- | --- |
| **MS-like baseline MRI (≥ 1 brain lesion)** | Banwell 2011 [50] | Prospective national cohort; 332 children with acute demyelination | Conversion | Diagnosis of MS | HR 37.9 (95% CI 5.26–273.85) | — |
| **Meeting MS diagnostic criteria at first MRI** | Mikaeloff 2006 [18] | Prospective; 197 POMS | Prognosis | Third attack; EDSS > 4 | HR 1.89 (95% CI 1.26–2.85), p = 0.01 (Cox model) | A |
| **Brainstem lesions** | De Meo 2021 [16] | Longitudinal cohort; 123 POMS | Prognosis | 9-year EDSS score | β = 0.31, p = 0.04 | A |
| **Cervical cord lesions** | De Meo 2021 [16] | Longitudinal cohort; 123 POMS | Prognosis | 9-year EDSS; ARR | 9-year EDSS: β = 0.22, p = 0.05. ARR: β = 0.16, p = 0.003 | A |
| **Optic nerve lesions** | De Meo 2021 [16]; Mikaeloff 2004 [26] | Longitudinal cohort (123 POMS); prospective cohort (296 first CNS demyelinating event) | Prognosis (relapse) / Conversion | Time to first relapse; second demyelinating attack | Time to first relapse: HR 2.10, p = 0.02 [16]. Second attack: HR 2.59, p = 0.009 [26] | A |
| **Cerebellar lesions** | De Meo 2021 [16] | Longitudinal cohort; 123 POMS | Prognosis | ARR | β = −0.15, p < 0.001 (lower relapse frequency) | B |
| **Corpus callosum perpendicular lesions** | Mikaeloff 2004 [26] | Prospective cohort; 296 children with first CNS demyelinating event | Conversion | Second demyelinating attack | Associated with increased risk of a second demyelinating attack | — |
| **≥ 2 new T2 lesions at 1–2 years** | De Meo 2021 [16] | Longitudinal cohort; 123 POMS | Prognosis | 9-year disability worsening; 9-year EDSS | 9-year worsening: OR 4.91, p = 0.02 (at 2 years). 9-year EDSS: β = 0.28 (1 year), p = 0.03; β = 0.35 (2 years), p = 0.01 | A |
| **Gd-enhancing brain / cervical cord lesions at year 1** | De Meo 2021 [16] | Longitudinal cohort; 123 POMS | Prognosis | 9-year EDSS | Cervical cord Gd+: β = −0.41, p = 0.02. Brain Gd+: β = −0.29, p = 0.05. Negative (i.e. apparently favourable) coefficient unexplained; plausibly reflects imaging timing relative to relapse and subsequent treatment escalation rather than protective inflammation | D |
| **Infratentorial lesion number** | Margoni 2026 [27] | Prospective multicentre; 52 POMS (12.3-year follow-up) | Prognosis | Time to first relapse | HR 1.09, p = 0.016 | B |
| **Spinal cord lesion presence** | Margoni 2026 [27] | Prospective multicentre; 52 POMS | Prognosis | Time to first relapse | HR 2.45, p = 0.017 | B |
| **Thalamic volume (lower)** | Margoni 2026 [27] | Prospective multicentre; 52 POMS | Prognosis | Time to first relapse | HR 0.77, p = 0.046 | B |
| **White matter lesion volume** | Margoni 2026 [27] | Prospective multicentre; 52 POMS | Prognosis | Recurrent relapse risk | HR 1.04, p = 0.013 | B |
| **NAWM fractional anisotropy (lower)** | Margoni 2026 [27] | Prospective multicentre; 52 POMS | Prognosis | 6-month confirmed disability worsening; EDSS worsening | Time to 6-month CDW: HR 0.67, p = 0.004. EDSS worsening: β = −0.26, p = 0.029 | B |
| **Reduced brain volume at presentation; brain growth failure** | Bartels 2019 [65] | Observational; pediatric MS vs controls | Prognosis (candidate) | Brain volume trajectory | POMS associated with reduced brain volumes at first clinical presentation and subsequent failure of age-expected brain growth | B |
| **Grey matter / posterior brain damage; network integrity** | Rocca 2014 [66]; Fuchs 2019 [67] | Observational; pediatric and adult MS | Prognosis (candidate) | Cognitive impairment; cognitive reserve | Posterior grey matter damage associated with cognitive impairment in pediatric MS [66]; preserved network functional connectivity underlies cognitive reserve [67] | B |

*ARR, annualised relapse rate; CDW, confirmed disability worsening; CNS, central nervous system; EDSS, Expanded Disability Status Scale; Gd+, gadolinium-enhancing; HR, hazard ratio; NAWM, normal-appearing white matter; OR, odds ratio; POMS, pediatric-onset multiple sclerosis. Advanced MRI metrics (thalamic volume, NAWM integrity) require pediatric normative reference values and harmonised acquisition protocols before they can be incorporated into a prognostic model.*

**Table S3. Fluid biomarkers**

| **Factor** | **Study [ref]** | **Design; N** | **Axis of inference** | **Outcome predicted** | **Effect estimate** | **Evidence tier** |
| --- | --- | --- | --- | --- | --- | --- |
| **Serum NfL — lesion burden and disease activity** | Reinert 2020 [29] | Retrospective cohort; 55 POMS | Prognosis | MRI lesion burden; relapse frequency; recent activity | Cerebral lesion load > 10 lesions: positive association, p < 0.001. Spinal cord lesions: p < 0.05. Number of relapses: p < 0.05. Untreated MS vs controls: median 19.0 vs 4.6 pg/mL, p < 0.001. Recent relapse (< 90 days): +51.1%, p < 0.001. Per contrast-enhancing lesion: +9.1%, p < 0.001 | A |
| **Serum NfL — treatment escalation** | Reinert 2020 [29] | Retrospective cohort; 55 POMS | Prognosis | Switch to high-efficacy therapy | OR 2.60, p = 0.024 | A |
| **Serum NfL — treatment response** | Reinert 2020 [29] | Retrospective cohort; 55 POMS | Prognosis (dynamic marker) | sNfL change on DMT | Interferon (6 ± 2 months): 14.7 → 7.9 pg/mL, p < 0.001. Fingolimod: 16.5 → 10.0 pg/mL, p < 0.001 | A |
| **Plasma NfL — baseline value** | Kuhle 2023 [31] | Post-hoc analysis of randomised trial (TERIKIDS) | Prognosis | Subsequent MRI or clinical activity | Each doubling of baseline NfL associated with a higher hazard of subsequent radiological or clinical activity | A |
| **Serum NfL — proximity to activity events** | Ziaei 2023 [30] | Retrospective; 142 POMS | Prognosis | Clinical / MRI disease activity | High sNfL levels observed close in time to disease activity events in POMS and MOGAD | A |
| **Serum NfL — mixed ADS cohorts** | Wendel 2022 [28] | Longitudinal prospective; 129 ADS | Prognosis (with caveat) | Disease activity across demyelinating phenotypes | sNfL elevated in MS and in monophasic MOGAD; estimates from mixed ADS cohorts may not transfer directly to confirmed POMS | B |
| **CSF oligoclonal bands** | Mikaeloff 2004 [26]; Banwell 2011 [50] | Prospective cohorts; 296 and 332 children with first demyelinating event | Conversion | Conversion to MS; subsequent disability | HR 6.33 (95% CI 3.35–11.96) for MS diagnosis [50]; linked to a more severe subsequent course [26] | — |
| **Serum GFAP** | Abdelhak 2022 [69]; Meier 2023 [70]; Saucier 2024 [71] | Adult MS cohorts; limited pediatric data | Prognosis (candidate) | Progression independent of relapse activity; chronic inflammatory burden | In adult MS, sGFAP is associated with progression independent of relapse activity, with a profile distinct from sNfL [69,70]. Pediatric data scarce [71]. Incremental value of sNfL + sGFAP over sNfL alone untested in children | B |
| **Chitinase-3-like protein 1 (CHI3L1)** | Comabella 2010 [72] | Adult CIS cohort | Conversion / Prognosis (candidate) | Conversion to MS; disability accrual | CSF CHI3L1 associated with conversion to MS and disability accrual in adults; limited pediatric data | B |
| **CSF kappa free light chains** | Konen 2021 [73] | Adult MS; methodological review | Conversion (candidate) | Diagnosis / conversion | Proposed as a robust, less operator-dependent alternative to oligoclonal bands; pediatric validation lacking | B |

*ADS, acquired demyelinating syndrome; CIS, clinically isolated syndrome; CSF, cerebrospinal fluid; DMT, disease-modifying therapy; GFAP, glial fibrillary acidic protein; HR, hazard ratio; MOGAD, myelin oligodendrocyte glycoprotein antibody-associated disease; NfL, neurofilament light chain; OR, odds ratio; POMS, pediatric-onset multiple sclerosis. Pediatric-specific caveats apply to sNfL: concentrations vary with age and body mass index (age-adjusted Z-scores required); Simoa and Ella platforms are not fully interchangeable; several pediatric cohorts comprise mixed acquired demyelinating syndromes.*

**Table S4. Treatment-related factors**

| **Factor** | **Study [ref]** | **Design; N** | **Axis of inference** | **Outcome predicted** | **Effect estimate** | **Evidence tier** |
| --- | --- | --- | --- | --- | --- | --- |
| **Delay in DMT initiation (per year)** | Kopp 2020 [17] | Prospective Danish registry cohort; 291 POMS | Prognosis | Sustained EDSS 4.0; confirmed EDSS improvement | Sustained EDSS 4.0: HR 1.17 (95% CI 1.05–1.30), p = 0.004. Confirmed improvement: HR 0.90 (95% CI 0.84–0.96), p = 0.001 | A |
| **Two-year delay vs early DMT** | Kopp 2020 [17] | Prospective Danish registry cohort; 291 POMS | Prognosis | Sustained EDSS 4.0; confirmed EDSS improvement | Sustained EDSS 4.0: HR 2.52 (95% CI 1.01–6.34), p = 0.049. Confirmed improvement: HR 0.39 (95% CI 0.26–0.59), p < 0.0001 | A |
| **Early high-efficacy therapy (HET) exposure** | De Meo 2021 [16] | Longitudinal observational cohort; 123 POMS | Prognosis | ARR at year 2 | β = −0.14, p = 0.01 (linear regression) | A |
| **HET exposure** | Margoni 2026 [27] | Prospective multicentre; 52 POMS (12.3-year follow-up) | Prognosis | Time to first relapse; recurrent relapse risk | Time to first relapse: HR 0.20, p = 0.010. Recurrent relapse risk: HR 0.21, p < 0.001 | A |
| **HET vs moderate-efficacy therapy** | Baroncini 2021 [55] | Italian MS Registry; multicentre real-world cohort | Prognosis | Disability worsening across disability states | HR 0.41 (95% CI 0.31–0.53) in the minimal-disability state | A |
| **DMT class and disability worsening** | Sharmin 2024 [56] | Longitudinal analysis of global and national registries (MSBase; Italian MS Registry) | Prognosis | Disability worsening | Concordant protective effect of high-efficacy therapy on disability worsening in POMS | A |
| **First-line HET** | Benallegue 2024 [57] | French national cohort (OFSEP) | Prognosis | Time to first relapse | 54% reduction in first-relapse risk: HR 0.46 (95% CI 0.31–0.67), p < 0.001 | A |
| **High-efficacy therapy — narrative synthesis** | Aljomah & Yeh 2025 [58] | Review | Prognosis | Long-term outcome | Narrative synthesis supporting improved outcome with high-efficacy therapies in POMS | A |

*ARR, annualised relapse rate; DMT, disease-modifying therapy; EDSS, Expanded Disability Status Scale; HET, high-efficacy therapy; HR, hazard ratio; POMS, pediatric-onset multiple sclerosis. Important caveat: all associations in this table are subject to confounding by indication. Children with more aggressive disease are escalated earliest to high-efficacy therapy, so the observational benefit of HET is confounded, and the apparent prognostic effect of adverse baseline features is attenuated in treated cohorts. Dynamic markers (sNfL, lesion accrual, relapse rate) change after treatment initiation. PARADIGMS (fingolimod vs interferon beta-1a) remains the only randomised comparison of a high-efficacy agent against an injectable in this population.*

**Table S5. Dietary, environmental, perinatal and parental factors**

*Unless otherwise indicated, the factors in this table pertain to the risk of developing POMS and have not been shown to predict relapse activity, disability accrual, cognitive decline or SPMS conversion among children already diagnosed with MS. They are reported for context and for their relevance to primary prevention, and are not proposed as candidate variables for a prognostic score. The three rows graded B were assessed against post-diagnosis outcomes and are therefore prognostic in nature.*

| **Factor** | **Study [ref]** | **Design; N** | **Axis of inference** | **Outcome predicted** | **Effect estimate** | **Evidence tier** |
| --- | --- | --- | --- | --- | --- | --- |
| **High BMI / obesity in childhood and adolescence** | Milles 2021 [32]; Chitnis 2016 [34]; Brenton 2019 [35]; Langer-Gould 2013 [36]; Hagman 2025 [37]; Papetti 2023 [38] | Case-control and cohort studies; 569–21,652 individuals | Susceptibility | Risk of POMS; age at onset | Boys with POMS: current BMI +2.9 and recalled BMI +2.95 vs controls, p < 0.01 [32]. Consistent association with greater MS risk and earlier onset across cohorts | C |
| **Low vitamin D (genetic risk score)** | Gianfrancesco 2017 [33] | Mendelian randomisation | Susceptibility | Risk of POMS | Vitamin D GRS associated with higher serum 25(OH)D decreased POMS odds: OR 0.72 (95% CI 0.55–0.94), p = 0.02. Mendelian randomisation design strengthens causal inference | C |
| **Vitamin D status after diagnosis** | Mowry 2010 [43]; Graves 2020 [42] | Observational cohorts; POMS | Prognosis | Relapse rate | Lower 25(OH)D associated with higher relapse rate in established POMS [43]; vitamin D pathway genes influence relapses [42]. Association, not causation; interventional data lacking | B |
| **Dietary intake (fat; saturated fat; vegetables)** | Azary 2018 [40] | Multicentre cohort; 219 POMS / pediatric CIS | Prognosis | Relapse rate | Each +10% energy from fat: +56% relapse risk. Each +10% saturated fat: threefold relapse risk. Each +1 cup-equivalent vegetable intake: −50% relapse risk. Self-reported intake, single cohort | B |
| **Dietary factors (case-control)** | Pakpoor 2018 [39] | Multicentre case-control | Susceptibility | Risk of POMS | Dietary factors associated with POMS risk. Note: the earlier claim regarding iron intake was not supported by these studies and has been removed from the main text | C |
| **Physical activity (lower)** | Grover 2015 [41] | Observational; POMS | Prognosis | Disease burden; lesion volume; relapse frequency | Lower physical activity associated with higher disease burden | B |
| **Passive smoke exposure** | Mikaeloff 2007 [45]; Lavery 2019 [46]; Pilotto 2026 [44] | Case-control and cohort studies (incl. PEDIGREE) | Susceptibility | Risk of POMS | Father smoking: crude OR 1.94 (95% CI 1.10–3.40). Both parents smoking pre-pregnancy: crude OR 10.79 (95% CI 1.30–89.54). Risk amplified in HLA-DRB1*15-positive children | C |
| **HLA-DRB1*15 allele** | Banwell 2011 [50] | Prospective national cohort; 332 children with acute demyelination | Susceptibility | Risk of MS diagnosis | HR 2.32 (95% CI 1.25–4.30) | C |
| **EBV and HSV-1 seropositivity** | Nourbakhsh 2018 [47]; Banwell 2011 [50] | Observational and prospective cohorts | Susceptibility | Risk of POMS | Prior EBV infection: HR 2.04 (95% CI 0.99–4.20) [50]; heterogeneous associations of remote herpesvirus infections [47] | C |
| **Lack of breastfeeding** | Brenton 2017 [48] | Cross-sectional case-control; 36 POMS | Susceptibility | Risk of POMS | Non-breastfed vs breastfed: OR range 1.68–11.71, p = 0.003 | C |
| **Caesarean delivery** | Graves 2017 [49] | Case-control; 265 POMS | Susceptibility | Risk of POMS | Reported as associated with a lower risk of POMS. Observational and subject to confounding by indication for delivery; the earlier description as 'protective' overstated the evidence | C |
| **Maternal illness during gestation** | Graves 2017 [49]; Banwell 2011 [50] | Case-control; prospective national cohort | Susceptibility | Risk of POMS | Approximately doubled odds of POMS onset | C |
| **Paternal occupational exposure (gardening, pesticide use)** | Banwell 2011 [50] | Prospective national cohort; 332 POMS | Susceptibility | Risk of POMS | Associated with increased disease risk | C |
| **Household chemical / pesticide exposure** | Mar 2018 [51] | Case-control; 312 POMS | Susceptibility | Risk of POMS | Several household chemical exposures associated with increased odds of POMS | C |
| **Air pollution (fine particulate matter; sulfur dioxide; lead; ozone)** | Mar 2018 [51]; Bergamaschi 2026 [52] | Case-control; PEDIGREE study (113 cases, 117 controls) | Susceptibility | Risk of POMS | Associated with increased odds of POMS. Ozone (O3) exposure: OR 1.11 (95% CI 1.03–1.19) in the first year before onset [52] | C |
| **Gut microbiota alterations** | Tremlett 2016 [53]; Horton 2021 [54] | Pilot and observational studies; POMS | Susceptibility / exploratory | Immune activation; MS activity | Associated with immune activation and MS activity in children. Evidence largely cross-sectional and exploratory; a consistent correlation with disease activity is not established | C |

*BMI, body mass index; CIS, clinically isolated syndrome; EBV, Epstein-Barr virus; GRS, genetic risk score; HLA, human leukocyte antigen; HR, hazard ratio; HSV-1, herpes simplex virus type 1; OR, odds ratio; POMS, pediatric-onset multiple sclerosis; SPMS, secondary progressive multiple sclerosis.*
